# Study on the Identification of *Radix Bupleuri* from Its Unofficial Varieties Based on Discrete Wavelet Transformation Feature Extraction of ATR-FTIR Spectroscopy Combined with Probability Neural Network

**DOI:** 10.1155/2015/950209

**Published:** 2015-02-15

**Authors:** Wenying Jin, Chayan Wan, Cungui Cheng

**Affiliations:** ^1^Department of Computer Engineering, Yiwu Industrial and Commercial College, Yiwu 322000, China; ^2^College of Chemistry and Life Science, Zhejiang Normal University, Yingbin Avenue 688, Jinhua 321004, China

## Abstract

The attenuated total reflection-Fourier transform infrared spectroscopy (ATR-FTIR) was employed to acquire the infrared spectra of *Radix Bupleuri* and its unofficial varieties: the root of *Bupleurum smithii* Wolff and the root of *Bupleurum bicaule* Helm. The infrared spectra and spectra of Fourier self-deconvolution (FSD), discrete wavelet transform (DWT), and probability neural network (PNN) of these species were analyzed. By the method of FSD, there were conspicuous differences of the infrared absorption peak intensity of different types between *Radix Bupleuri* and its unofficial varieties. But it is hard to tell the differences between the root of *Bupleurum smithii* Wolff and the root of *Bupleurum bicaule*. The differences could be shown more clearly when the DWT was used. The research result shows that by the DWT technology it is easier to identify *Radix Bupleuri* from its unofficial varieties the root of *Bupleurum smithii* Wolff and the root of *Bupleurum bicaule*.

## 1. Introduction

*Radix Bupleuri,* one of the famous Chinese herbs, plays an important role in human disease prevention and treatment and is widely used in Southeast Asia [1]. China, which is the birthplace of Chinese herbal medicine, has abundant medical resources. The main ingredients of the* Radix Bupleuri* includes carbohydrate, sitosterol, saikosaponin yuan F,* Bupleurum* times saponins F, 3′′-O-acetyl saikosaponin-D, 6′′-O-acetyl saikosaponin-D, 4′′-O-acetyl saikosaponin-D, and *α*′′-spinasterol twenty-four carbonate, and so forth [2]. In the herbal medicine market, it could be difficult to determine the authenticity of Chinese medicine, for example, the* Radix Bupleuri* and its unofficial varieties: the root of* Bupleurum smithii* Wolff and the root of* Bupleurum bicaule* Helm. They belong to the same family but different species, but the market place Medicine Inspection Department always found such phenomenon that these unofficial varieties came out: the root of* Bupleurum smithii* Wolff and the root of* Bupleurum bicaule* Helm are often sold as the* Radix Bupleuri*. They may have the same morphological characteristic, but they are completely different. For modernization and globalization of Chinese medicine, one key to success is to build a traditional Chinese medicine quality standard system [3]. The recent research focused on the application of modern instrumentation technology in combination with a comprehensive analysis [4–6]. Recently, the Fourier transform infrared spectroscopy (FT-IR) has become a sophisticated analytical tool in the characterisation of organic compounds. For example, the FT-IR method was used in the analysis of rats pancreatic cancer data [7]. As an analytical tool, infrared spectroscopy has many advantages. FT-IR spectroscopy is a spectroscopic technique based on the absorption of infrared photons that excite vibrations of molecular bonds. A spectrum of characteristic bands is produced that can be used as a fingerprint to identify and characterize the Chinese herbs [8]. The Fourier self-convolution (FSD) analysis technology and wavelet transform (WT) are a chemometrics method that can distinguish the images effectively. FSD can increase the resolution rate, so that we can analyze the overlapping peaks of the complex system more clearly [9]. Wavelet transformation is a more effective signal processing method than Fourier transform, and the transformed results (wavelet factor) of discrete wavelet transform (DWT) contain more valuable information, which is a relatively effective analysis method in chemometrics [10]. A probability neural network (PNN) is a mathematical model or computational model that is inspired by the structure and/or functional aspects of biological neural networks. PNN, with their remarkable ability to derive meaning from complicated or imprecise data, can be used to extract patterns and detect trends that are too complex to be noticed by either humans or other computer techniques. This technique has a very wide application in lots of classification and recognition areas [11–13]. This paper applies the wavelet feature extraction of the FT-IR data and PNN classification to distinguish* Radix Bupleuri* from its unofficial varieties the root of the* Bupleurum smithii* Wolff and the root of the* Bupleurum bicaule* Helm.

## 2. Materials and Methods

### 2.1. Apparatus

FT-IR spectra have been collected in Nicolet NEXUS 670 FT-IR spectrophotometer (Nicolet, Madison, WI) equipped with DTGS detector in the region 4000~650 cm^−1^ with fully computerized data storage. All data are processed with OMNIC E.S.P. 5.1 software, in which the ATR accessory was used. For all spectra reported a 64-scan data accumulation was used at a resolution of 2 cm^−1^ in transmission mode. Air background FT-IR spectrum is collected before collecting sample's FT-IR spectrum. Samples were collected directly by FT-IR with ATR.

### 2.2. Materials

The* Radix Bupleuri* belongs to the dried root of Umbelliferae Angustifolia* Bupleurum*; the* Bupleurum smithii* Wolff belongs to the dried root of Umbelliferae black* Bupleurum*; the* Bupleurum smithii* Helm belongs to the dried root of Umbelliferae cone leaves* Bupleurum*. All the samples are provided by Jinhua Food and Drug Institution of Zhejiang Province. There are 160 samples, respectively. All the samples were identified by Dr. Jianhua Chen from Department of Biology, College of Chemistry and Life Science, Zhejiang Normal University, China.

Each sample is first dried in an oven at 60°C for 72 hours. Afterwards, all the samples are grounded into a uniform fine powder on the agate mortar. Weigh 8.0 mg of sample accurately, which is used for testing.

### 2.3. Spectral Measurements

8.0 mg of predisposed samples was, respectively, placed directly about 3.14 mm^2^ on the center of the diamond crystal of the ATR accessory for measurement. To ensure good contact with the diamond crystal surface, all powder samples were pressed using a pressure tower to provide the same mechanical pressure on all samples. All obtained spectra were autobaseline corrected. No other sample preparation was required.

The FT-IR spectrum background was recorded before collecting the sample's FT-IR spectrum. Reference spectra were recorded using a blank diamond-ATR. Single beam spectra were obtained for all the samples and the ratio of the spectra against the background spectra of air was used to present the spectra in absorbance units. After each experiment the diamond-ATR was thoroughly washed with absolute ethyl alcohol and dried with nitrogen, and its spectra were examined to ensure that no residue from the previous experiment was retained on the diamond surface. All of the tissue samples dried with absorbent cotton and further dried with nitrogen. Each species of all samples were measured three times and the averaged spectrum was used for further analysis.

### 2.4. Data Analysis

The FT-IR of all the samples was obtained by measuring. According to the absorbance value characteristic of absorption peak, we can make the Fourier self-deconvolution (FSD) analysis to the data. NEXUS E.S.P. 5.2 software was used for this analysis. The absorption values from different wave bands based on the characters of the absorption value were obtained by copying data method (export the FT-IR data as coordinate value, then copy the data). MatLab 6.5 software was used for discrete wavelet transformation analysis. Daubechies wavelet which possesses better exploration ability for signal singularity acted as analysis wavelet and some scale one-dimension discrete wavelets from different samples were transformed. 5 layers of the samples character variable were picked up by selecting a decomposition scale whose difference degree was the most obvious. Through a comparative analysis, two layers (3 and 4) were selected to extract eigenvector. The selected character variables were used for PNN training and verification.

## 3. Results and Discussion

### 3.1. FT-IR Analysis

**Figure 1:**
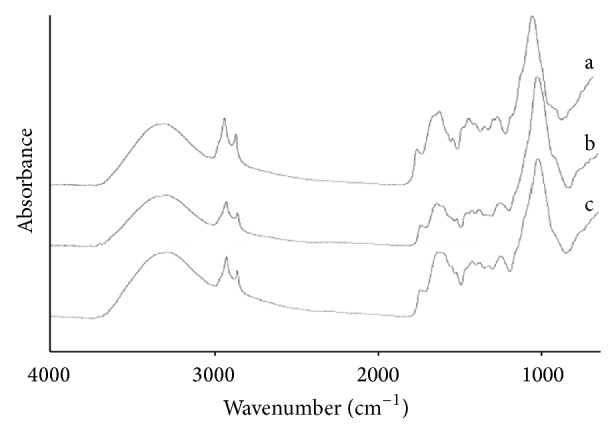
FT-IR spectra of the* Radix Bupleuri* and its unofficial varieties. a.* Radix Bupleuri*; b. root of the* Bupleurum smithii* Wolff; c. root of the* Bupleurum bicaule* Helm.

Figure 1 shows the typical FT-IR spectra of the* Radix Bupleuri*, the root of the* Bupleurum smithii* Wolff, and the root of the* Bupleurum bicaule* Helm.

From Figure 1, we notice that the* Radix Bupleuri*, the root of the* Bupleurum smithii* Wolff, and the root of the* Bupleurum bicaule* Helm, generated large numbers of sharp peaks in the FT-IR spectra region (4,000–650 cm^−1^). Absorption bands located around 3300 cm^−1^ correspond to O-H stretching vibrations and absorption bands located around 1030~1200 cm^−1^ correspond to C-O stretching vibrations that mainly occur from carbohydrates, which indicates these three samples contain lots of similar chemical compositions like cellulose carbohydrates. Though they have different populations and different growing environments, they are from the same family. Therefore, they have very closed absorbance and they are difficult to distinguish by experience. So the FSD and DWT methods were used for further classification.

### 3.2. Fourier Self-Deconvolution Infrared Spectroscopy Analysis

**Figure 2:**
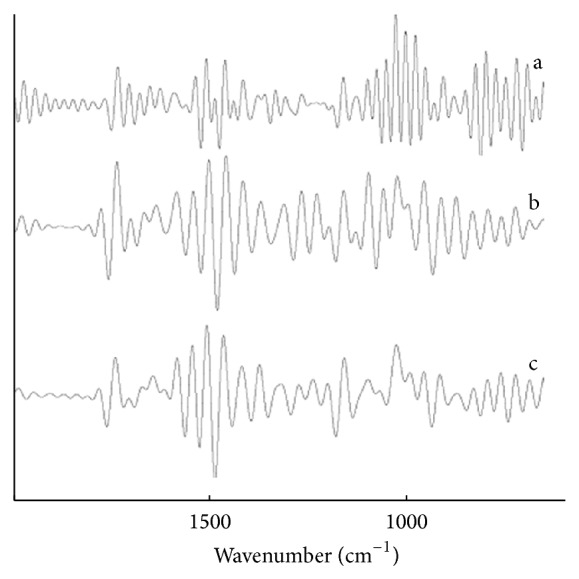
FSD-IR spectra of the* Radix Bupleuri* and its unofficial varieties. a.* Radix Bupleuri*; b. root of the* Bupleurum smithii* Wolff; c. root of the* Bupleurum bicaule* Helm.

FSD is a kind of technology for the peak separation of Fourier transformed overlapping peaks. It narrows peak shape of the IR absorption spectrum without changing peak position or area. The FSD methods could make a further analysis to the IR of the* Radix Bupleuri*, the root of the* Bupleurum smithii* Wolff, and the root of the* Bupleurum bicaule* Helm. Considering the main absorption under the 2000–4000 cm^−1^ region correspond to O-H, N-H stretching vibrations and saturated and unsaturated C-H stretching vibrations. Because the samples belong to the roots, therefore we selected the 2000–650 cm^−1^ region as the FSD analysis. When choosing the enhancement factor as 3.5, the bandwidth factor as 77.0, FSD method is used to the FT-IR of the* Radix Bupleuri*, the root of the* Bupleurum smithii* Wolff, and the root of the* Bupleurum bicaule* Helm. The results are shown in Figure 2.

From Figure 2, we can easily identify the* Radix Bupleuri*; the difference between the* Radix Bupleuri* and the other two* Bupleuris* is quite obvious. But as to the root of the* Bupleurum smithii* Wolff and the root of the* Bupleurum bicaule* Helm, they are a little similar, so we use the discrete wavelet feature extraction to the FT-IR of the three samples, using the probabilistic neural network as a further classification and identification.

### 3.3. Discrete Wavelet Feature Extraction

When we use the wavelet transformation to analyze data, proper wavelet basis function and decomposing level number should be determined according to the spectral characteristics of the signal. The suitable wavelet base and wavelet scale are determined by the effect of signal decomposition in different scales and the characteristics of the FT-IR signal in wavelet multidetail decomposition procedure. There is not a general criterion about how to choose the optimal wavelet basis function. In general, we choose a proper wavelet basis function by considering the properties of the wavelet basis function, features of signal to be analyzed, and actual problem. The part of the signal whose shape is similar to that of the wavelet basis function will be enlarged, and other parts of the signal will be suppressed. In addition, proper scale wavelet is used according to the real problems. Big scale wavelet basis function should be used if we describe the total and approximate properties of the signal by the wavelet transformation. Small scale wavelet basis function should be used if we extrude the scales of the signal by the wavelet transformation.

We will use the DWT to detect the singularity of the curvature curve, so we should choose proper wavelet, which has similar shape to the absorption peak analyzed, short compact branch set, and big vanishing moment, as wavelet basis function. Some representative wavelet basis functions include Mexihat, Meyer, Morlet, Daubechies, Coilet, and Symlets. Compared to other five wavelets, Daubechies wavelet has the shortest compact branch set, so we choose Daubechies wavelet as analyzing wavelet.

**Figure 3:**
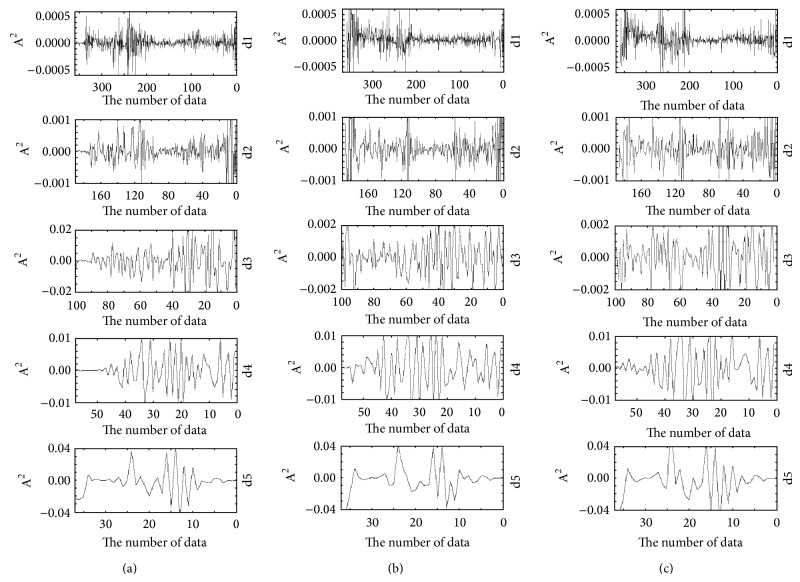
DWT of the* Radix Bupleuri* and its unofficial varieties. (a)* Radix Bupleuri*; (b) root of the* Bupleurum smithii* Wolff; (c) root of the* Bupleurum bicaule* Helm.

In this paper, the discrete wavelet transformation is done to the FT-IR spectra of the* Radix Bupleuri*, the root of the* Bupleurum smithii* Wolff, and the root of the* Bupleurum bicaule* Helm, respectively.  Figure 3 represents the DWT coefficients at five scales.

**Figure 4:**
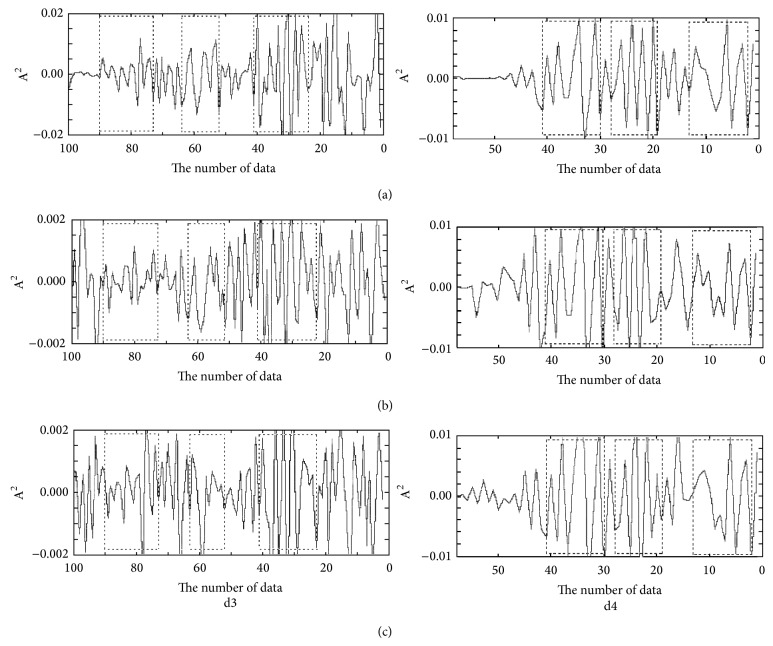
Division of three feature regions of detail signal in DWT domain. (a)* Radix Bupleuri*; (b) root of the* Bupleurum smithii* Wolff; (c) root of the* Bupleurum bicaule* Helm.

The approximation holds the low frequency components, and the detail holds high frequency components. Even the 5th scale approximation looks very similar to the original FT-IR data, but it is smoother than the original FT-IR after noise is removed. We choose two representative scales (details 3 and 4) to extract their characteristics. Characteristic variable is defined as the energy (wavelet coefficient squares) of spectrum at detail 3 and detail 4 in the DWT.  Figure 4 shows a diagram of a divided feature space. Three feature bands were selected from each detailed signal, which corresponds to the same feature peaks. The feature vectors were defined as the average value of energy at each feature band. Thus, nine feature variances were generated from three detailed signals.

### 3.4. The Methods of PNN Classification

**Figure 5:**
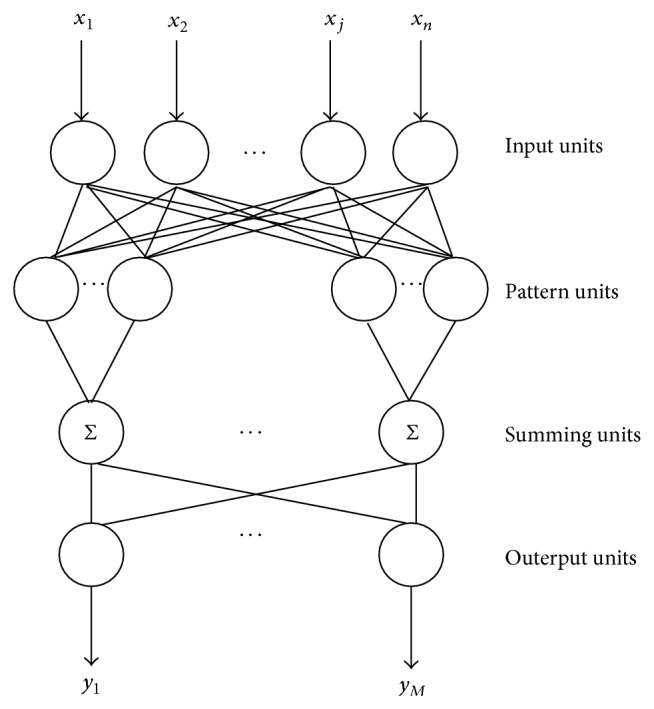
The topological structure of PNN.

PNN is the feed-forward network model of artificial neural network based on the theory of statistics with Parzen window function as the activation function. PNN absorbs the advantages of RBF neural network and classical theory of probability density estimation and compares with the traditional feed-forward neural network, especially has the remarkable advantage in the pattern classification aspect.  Figure 5 shows the topological structure of PNN.

The first and last layout, respectively, denotes input layout and output layout; the two middle layouts are hidden layouts.

To the input vector *x*, output value *Y*_*j*_ of the *i*th nerve cell in the output layout of PNN can be written as

(1) $$\begin{array}{c} Y_{j}=\overset{M}{\underset{k=1}{\sum }}w_{jk}H_{k}\left(x\right)\;j=1,2,\ldots ,M\\ H_{k(x)}=\overset{n_{k}}{\underset{i=1}{\sum }}P_{i}\left(\left\|x-c_{kj}\right\|\right), \end{array}$$

where *x* is input vector with dimension; *H*_*k*(*x*)_ is output of the *k*th unit in the second hidden layout; *w*_*jk*_ is connected weight between the *j*th nerve cell in the second hidden layout and the *k*th nerve cell of output layout; *P*(^*^) is Parzen window function; *c*_*kj*_ is the *k*th center vector of the *j*th class in the first hidden layout; *n*_*k*_ is the number of the center vectors of the *k*th class in the first hidden layout; ‖·‖ is euclidean norm; and *M* is the number of nerve cells in the output layout

### 3.5. The Results of PNN Classification

Through the PNN method, the three different types of samples (the* Radix Bupleuri,* the root of the* Bupleurum smithii* Wolff, and the root of the* Bupleurum bicaule* Helm) are correctly identified. The eigenvectors of the different samples that were extracted from the wavelet transform of the FT-IR have significant difference so that the high accuracy of the classification can be achieved. In this PNN program, selecting 180 samples divided into three groups as training samples; each group includes 60 samples. 300 test samples are divided into three as well. The results showed that it is 100% for identifying the* Radix Bupleuri* samples, while being 99% for identifying the root of the* Bupleurum smithii* Wolff and the root of the* Bupleurum bicaule* Helm, respectively.

## 4. Conclusion

The application of wavelet transformation and PNN in analytical chemistry is currently a very active field of research; direct determination of plant root samples by ATR-FTIR is convenient and fast. The proposed method has a high recognition rate to the* Radix Bupleuri* and its unofficial root of the* Bupleurum smithii* Wolff and the root of the* Bupleurum bicaule* Helm by combining probabilistic neural network with the DWT features of FT-IR of samples. Wavelet transformation combined with the PNN made them attractive in many applications. Given the activity in this field, we can expect much progress in the future.
